# Pupillary responses in non-proliferative diabetic retinopathy

**DOI:** 10.1038/srep44987

**Published:** 2017-03-23

**Authors:** Jason C. Park, Yi-Fan Chen, Norman P. Blair, Felix Y. Chau, Jennifer I. Lim, Yannek I. Leiderman, Mahnaz Shahidi, J. Jason McAnany

**Affiliations:** 1 Department of Ophthalmology and Visual Sciences, University of Illinois at Chicago, 1855 W. Taylor St., Chicago, IL 60612, USA; 2 Center for Clinical and Translational Sciences, University of Illinois at Chicago, 914 S Wood Street, Chicago, IL 60612, USA; 3 Department of Ophthalmology, University of Southern California, 1450 San Pablo St, Los Angeles, CA 90033, USA

## Abstract

The goal of this study was to determine the extent of rod-, cone-, and melanopsin-mediated pupillary light reflex (PLR) abnormalities in diabetic patients who have non-proliferative diabetic retinopathy (NPDR). Fifty diabetic subjects who have different stages of NPDR and 25 age-equivalent, non-diabetic controls participated. PLRs were measured in response to full-field, brief-flash stimuli under conditions that target the rod, cone, and intrinsically-photosensitive (melanopsin) retinal ganglion cell pathways. Pupil responses were compared among the subjects groups using age-corrected linear mixed models. Compared to control, the mean baseline pupil diameters were significantly smaller for all patient groups in the dark (all p < 0.001) and for the moderate-severe NPDR group in the light (p = 0.003). Pairwise comparisons indicated: (1) the mean melanopsin-mediated PLR was significantly reduced in the mild and moderate-severe groups (both p < 0.001); (2) the mean cone-mediated PLR was reduced significantly in the moderate-severe group (p = 0.008); (3) no significant differences in the mean rod-mediated responses. The data indicate abnormalities in NPDR patients under conditions that separately assess pupil function driven by different photoreceptor classes. The results provide evidence for compromised neural function in these patients and provide a promising approach for quantifying their neural abnormalities.

Diabetic retinopathy (DR) is the most serious ocular complication of diabetes mellitus and is the leading cause of new cases of legal blindness among adults between the ages of 20 and 74 years in the United States^1^. Current standards recommend the classification of DR stage based on the severity of clinically-apparent vascular abnormalities^2^. However, there is mounting evidence supporting neural dysfunction, even at early disease stages, in addition to the well known retinal vasculature abnormalities in these patients (see Leith, *et al*.^3^ and Adams and Bearse^4^ for reviews). For example, the steady-state pupil size has long been recognized to be abnormally small in diabetics, e.g. ref. 5 a finding attributed to abnormal sympathetic nervous system innervation^6^,^7^,^8^. Additionally, in an exploratory case series, some patients who had type 2 diabetes and no clinically-apparent retinopathy had abnormal pupil responses elicited by brief light stimulation, a finding attributed to impaired function of the neural retina^9^.

Relatively recent advances in the understanding of the neural mechanisms that mediate the pupil response have greatly renewed interest in pupillometry as a tool for assessing retinal function in patients with acquired^9^,^10^,^11^ and inherited^12^,^13^,^14^,^15^ retinal disease. That is, the afferent limb of the pupillary response to light is now thought to be driven primarily by intrinsically photosensitive retinal ganglion cells (ipRGCs) that contain the photopigment melanopsin^16^,^17^. These ipRGCs are a third class of photoreceptor, distinct from rods and cones. Despite constituting only a small fraction of the total RGC population (approximately 0.2% in the primate retina^16^), these cells largely control pupil size^18^. The pupillary response can be used as an index of ipRGC function, which in turn may provide insight into inner-retina dysfunction in diabetic patients.

Although the ipRGCs are directly sensitive to light, they also receive input from rod and cone photoreceptors^16^,^19^,^20^. As such, the response of the pupil to a flash of light can be complex, with potential contributions from rods, cones, and ipRGCs^21^,^22^,^23^. However, by altering the adaptation conditions and stimulus characteristics, both inner- and outer-retinal contributions to the pupil response can be examined^14^,^15^,^24^,^25^. The pupil response driven by inner-retina neurons (melanopsin-mediated response) is characterized by a prolonged constriction following the offset of the stimulus, whereas the rod- and cone-mediated responses are characterized by rapid, transient constrictions^15^,^26^,^27^,^28^. To date, previous studies have focused primarily on the melanopsin-mediated response (often referred to as the post-illumination pupil response; “PIPR”) and pupillary responses have not been reported under rod- and cone-mediated conditions in diabetic patients. However, these measurements would be of value given the reports of potential inner-retina^29^,^30^,^31^ and outer-retina^32^,^33^ neural abnormalities in these individuals.

In the current study, an established protocol^15^,^24^,^34^ was used to evaluate rod-, cone-, and melanopsin-mediated pupil responses in diabetic patients who have different stages of non-proliferative DR (NPDR). Pupil responses were also measured in visually-normal, non-diabetic control subjects and in diabetic patients who have no clinically-apparent retinopathy. Our goal was to determine the effects of diabetes on the various pupillary measures and to evaluate the relationship between the pupillary response and disease stage. The pupil measurements were also compared to patient characteristics such as HbA1C percentage, diabetes duration, age, and sex.

## Methods

### Subjects

Fifty subjects diagnosed with type-2 diabetes mellitus and 25 visually-normal, non-diabetic control subjects were recruited from the University of Illinois Hospital and Health Sciences System. For all subjects, a comprehensive history was obtained from the medical record and a physical examination of each eye was performed by a retina specialist (authors NB, FC, JL, or YL) with particular attention to the optic nerve, retina, and its vasculature. Diabetic subjects had best corrected visual acuity of 0.4 log MAR or better (Snellen equivalent of approximately 20/50 or better) and controls had visual acuity of 0.1 log MAR or better (Snellen equivalent of approximately 20/25 or better). Subjects were excluded if there was a history of, or if they presented with, ischemic optic neuropathy, other optic nerve disease, retinal artery occlusion, retinal vein occlusion, or glaucoma. Additionally, subjects with iris neovascularization, iris atrophy, or an asymmetrically shaped pupil were excluded. No subject had a history of neurologic events (cerebrovascular stroke or transient ischemic attack). The lens of each subject was graded by slit lamp examination using a subjective clinical scale that ranged from clear to 4+. Subjects with nuclear sclerotic, posterior subcapsular, or cortical lens opacities greater than 2+ were excluded. Although subjects with lens opacities of 2+ or less were included, we note that the effects of light scatter due to lens opacities are negligible because the stimuli were uniform full-field flashes. All 25 control subjects and 41 of the 50 patients were phakic; the remaining 9 patients were pseudophakic. No subject had cataract surgery in the 9 months prior to testing.

For the diabetic subjects, the stage of NPDR was graded and the subjects were classified as diabetic with no NPDR (N = 17), mild NPDR (N = 16), moderate NPDR (N = 13), and severe NPDR (N = 4), based on the International Clinical Disease Severity Scale for DR^2^. Given the small number of subjects with severe NPDR, these subjects were grouped with those who had moderate NPDR for subsequent analysis. Subject characteristics including age, sex, estimated diabetes duration, HbA1c percentage, and treatment history are provided in Table 1. Seven patients who had mild NPDR and 13 patients who had moderate-severe NPDR also had a history of clinically-significant diabetic macular edema. These patients received focal laser treatment and/or intravitreal injection of anti-VEGF agents not less than 9 month prior to testing. Three patients who had mild NPDR and 4 patients who had moderate-severe NPDR had a history of both focal laser and intravitreal injection of anti-VEGF agents. The mean age of the subjects within the various groups did not differ significantly (F = 1.41, p = 0.24). The research followed the tenets of the Declaration of Helsinki and was approved by an institutional review board of the University of Illinois at Chicago. All subjects provided written informed consent.

### Apparatus, stimuli, and procedure

An LED-driven ganzfeld system was used for stimulus generation and display (Espion V6; ColorDome desktop ganzfeld, Diagnosys LLC, Lowell, MA). Details and images of the system are available elsewhere^28^,^34^. Stimulus presentation and pupil recordings were performed monocularly using a ViewPoint EyeTrack infrared camera system (Arrington Research, Scottsdale, AZ), with the fellow eye patched. The infrared camera system measured the pupil diameter at a 60 Hz sampling frequency. Data were typically obtained from the right eye of all subjects. However, in rare cases in which the NPDR stage differed between the eyes, the eye with the lower NPDR stage was tested, as we were interested in the early effects of diabetes on the pupil response. Test protocols intended to target the rod, cone, and melanopsin pathways were performed, as described in detail elsewhere^15^,^24^. In brief, subjects were tested with the following three paradigms: (1) under the rod paradigm, a short-wavelength (“blue;” dominant wavelength of 465 nm), low luminance (0.001 cd/m^2^) flash was presented in the dark; (2) under the melanopsin paradigm, a short-wavelength (“blue;” dominant wavelength of 465 nm), high luminance (450 cd/m^2^) flash was presented in the dark; (3) under the cone paradigm, a long-wavelength (“red;” dominant wavelength of 642 nm), moderate luminance (10 cd/m^2^) flash was presented against a rod-suppressing short-wavelength adapting field (6 cd/m^2^). All flashes were 1 sec in duration. Responses to a minimum of two flashes (separated by at least 15 sec for the rod and cone conditions and 60 sec for the melanopsin condition) were obtained and averaged for analysis. Prior to the stimulus presentation under the rod and melanopsin paradigms, the subjects were dark-adapted for 10 minutes, whereas the subjects were light adapted for 2 minutes to the rod-suppressing adapting field prior to stimulus presentation under the cone paradigm. Stimulus wavelength and luminance were verified with a spectroradiometer (SpectraScan 740, Photo Research, Chatsworth, CA).

### Data analysis

Data were analyzed using custom scripts programmed in MATLAB (MathWorks Inc., Natick, MA), which allowed for semi-automated analysis as described elsewhere^15^,^24^. In brief, the response of the pupil to a flash of light (the pupillary light reflex; PLR) was normalized by the median steady-state (baseline) pupil size during the 1 sec preceding each stimulus onset. Normalization to baseline helps to minimize the effects of inter-subject differences in the baseline pupil size. Two measures were derived: 1) under the rod- and cone-mediated conditions, the normalized transient amplitude was used as the PLR measure. The transient PLR amplitude was defined as the difference between the normalized baseline and the minimum normalized PLR after stimulus onset (maximum constriction); (2) under the melanopsin-mediated condition, the sustained response (“PIPR”) was used. The sustained response was defined as the difference between the normalized baseline and the median normalized PLR measured over a 5 to 7 sec time range following stimulus offset.

Pupil size was compared among the subject groups statistically using linear mixed models to account for covariates^35^,^36^,^37^. One set of models compared baseline pupil size among the subject groups (control, no NPDR, mild NPDR, moderate-severe NPDR). A second set of models compared the flash-driven PLRs among the subject groups, measured under melanopsin-, rod-, and cone-mediated conditions, after accounting for baseline pupil size. All models were adjusted for subject age and included a random intercept at the subject level to account for possible subject dependencies (due to the repeated measures design). All pairwise comparisons among subject groups were performed using least-squares mean differences with Tukey adjustment. Correlation analyses were performed by computing Pearson’s (or Spearman’s) correlation coefficients; Bonferroni correction was used to account for multiple correlations. All statistical analyses were performed using Sigmaplot software version 12.0 (Systat Software, Inc, San Jose, CA) and R (R Foundation for Statistical Computing, Vienna, Austria).

## Results

**Figure 1:**
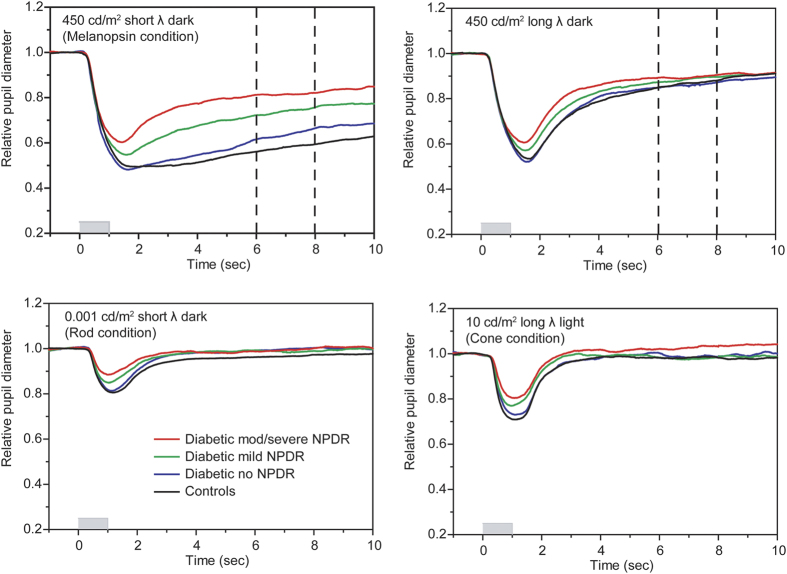
Mean waveforms obtained under the melanopsin (top left), rod (lower left), and cone (lower right) paradigms stratified by subject group. The upper right panel shows responses measured with a long wavelength flash (450 cd/m^2^) presented in the dark, for comparison to the melanopsin-mediated condition. The long wavelength 450 cd/m^2^ flash is not typically included in the standard set of conditions (rod, cone, melanopsin) and data from this condition will not be considered further. The red traces represent the mean waveform for the moderate-severe NPDR group, the green traces represent the mean waveform for the mild NPDR group, the blue traces represent the mean waveform for the no NPDR group, and the black traces represent the mean waveform for the control group. The vertical dashed lines in the top panels indicate the range over which the melanopsin-mediated (sustained) amplitude was measured. The stimulus onset and offset are represented by the boxes along the x-axes.

Figure 1 shows the mean normalized pupil traces from each subject group. PLRs were obtained under the melanopsin- (top left), rod- (lower left), and cone-mediated (lower right) conditions. The mean control (black trace) PLR obtained under the melanopsin condition is characterized by an initial transient constriction (rod- and cone-mediated) followed by a sustained constriction that persists for several seconds after stimulus offset (melanopsin-mediated). The initial transient constriction was somewhat reduced in the mild and moderate-severe NPDR groups. The initial transient constriction and the early re-dilation phase have been shown to contain both rod and melanopsin contributions^38^. Importantly, the mean pupil response for each patient group under the melanopsin condition had a return towards baseline that was more rapid than that observed for the control group. This resulted in a mean melanopsin-mediated PLR for each patient group that was smaller than the control mean over the period for which the sustained PLR was measured (vertical dashed lines). The PLR elicited by a long-wavelength flash of the same luminance (450 cd/m^2^) presented in the dark is shown in the upper right panel for comparison to the short-wavelength flash used under the melanopsin condition. The response elicited by the long wavelength flash consisted of only the initial transient constriction, which was somewhat reduced in the mild and moderate-severe NPDR groups. For the long wavelength flash, there was no sustained response for any subject group and the traces for the control and patient groups are nearly superimposed in the 5 to 7 sec window following stimulus offset (vertical dashed lines). Since a substantial sustained PLR was not obtained with the long wavelength flash, data from this condition will not be considered further.

For the rod (Fig. 1, lower left) and cone (Fig. 1, lower right) conditions, the normal PLR is characterized by a rapid transient constriction (peak latency <2 sec) followed by a relatively rapid return to the baseline. Although the PLRs of the patients were similar in shape to that of the controls, the transient responses (i.e. maximum constrictions) were reduced for patients in the mild and moderate-severe groups. The PLR traces shown in Fig. 1 are intended to provide examples of the effect of light stimulation on the pupil response measured under the melanopsin-, rod-, and cone-mediated conditions; individual response amplitudes derived from the PLR traces for all patients and controls are evaluated quantitatively below.

**Figure 2:**
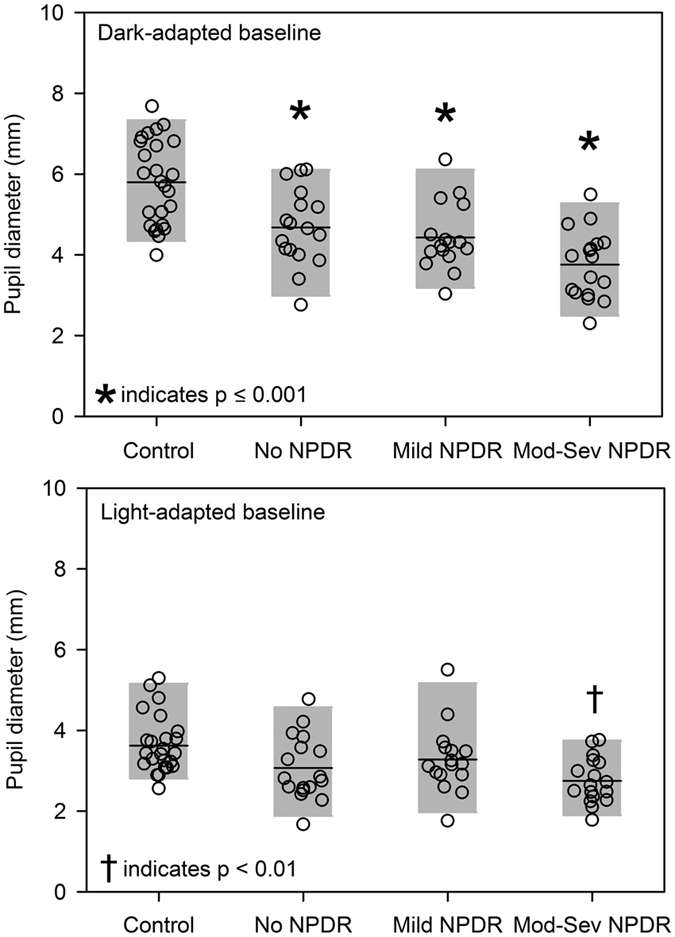
Steady-state (baseline) pupil diameters for each subject measured under dark-adapted (top) and light-adapted (bottom) conditions. The horizontal line represents the mean for each group and the gray region represents the 5^th^ to 95^th^ percentile of each group. Significant differences from the control group are indicated by the symbols above the groups.

Figure 2 shows the steady state (baseline) pupil size under dark-adapted (Fig. 2, top) and light-adapted (Fig. 2, bottom) conditions. Data are shown for each control subject (leftmost data set) and for each patient with diabetes (stage progresses from left to right). The dark-adapted baseline pupil size decreased systematically across disease stage (Spearman’s r = −0.40, p = 0.004), with moderate-severe NPDR patients having a baseline pupil size that was reduced by 37% on average compared to controls. Interestingly, even diabetics with no clinically-apparent retinopathy had, on average, a baseline pupil size that was smaller than normal. Age-adjusted, Tukey pairwise comparisons indicated significantly smaller dark-adapted baseline pupil sizes for all three patient groups compared to the control group (all t > 3.94, all p ≤ 0.001).

In general, a similar pattern in baseline pupil diameter was observed under the light-adapted condition, but the baseline abnormalities were smaller than those observed under dark-adapted conditions. The correlation between the light-adapted baseline pupil size and disease stage was not statistically significant (Spearman’s r = −0.15, p = 0.31). Age adjusted, Tukey pairwise comparisons indicated significantly smaller light-adapted baseline pupil sizes only for the patients with moderate-severe NPDR compared to the control group (t = 3.63, p = 0.003). The mean (±SEM) dark- and light-adapted age-adjusted baseline pupil sizes (mm) for each group are given in Table 2 (first two rows).

As an additional consideration, several patients in the mild and moderate-severe NPDR groups had a history of treatment with focal laser therapy and/or anti-VEGF agents (see Table 1). To evaluate the effects of treatment, sub-analyses were performed after excluding the 8 patients who had focal laser therapy (Table 2; rows 3 and 4) and after excluding the 19 patients who had received anti-VEGF therapy (Table 2; rows 5 and 6). Table 2 (rows 3 to 6) indicates that excluding patients who had a history of treatment had negligible effects on the results. Specifically, the results of the sub-analyses were highly similar to the results based on the complete sample, with the exception that excluding patients who had moderate-severe NPDR and a history of anti-VEGF treatment resulted in a non-statistically significant difference from control for the light-adapted baseline (Table 2; row 6). However, this effect is likely due to the small sample size (N = 5), as approximately 71% of this group had a history of anti-VEGF therapy and were excluded from the sub-analysis.

**Figure 3:**
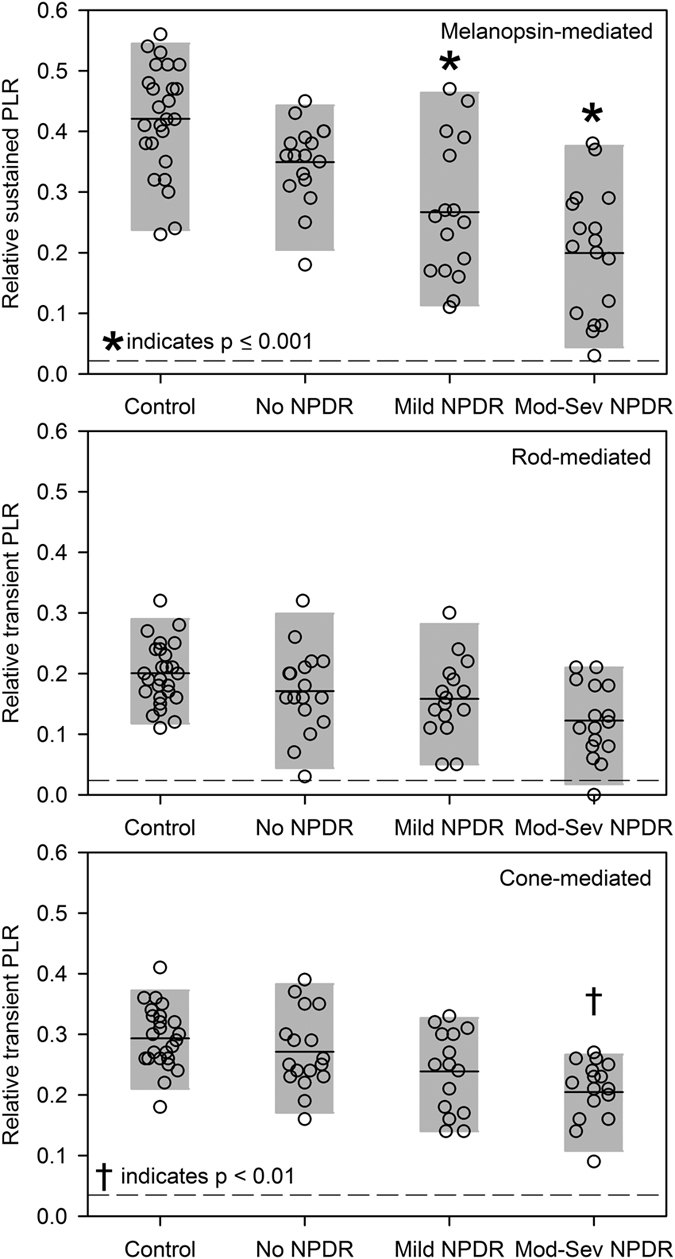
Relative PLR amplitudes obtained under the melanopsin (top), rod (middle), and cone (bottom) paradigms. The horizontal solid line represents the mean for each group and the gray region represents the 5^th^ to 95^th^ percentile of each group. Significant differences from the control group are indicated by the symbols above the groups and the horizontal dashed lines represent the noise level.

Figure 3 shows the relative melanopsin-mediated (sustained) PLR (Fig. 3, top), as well as the transient rod-mediated (Fig. 3, middle), and transient cone-mediated (Fig. 3, bottom) PLRs. The dashed horizontal lines indicate the noise level estimated from the baseline response for each condition; relative PLR amplitudes at or below this line can be considered extinguished. The melanopsin-mediated PLR amplitude was correlated significantly with disease stage (Spearman’s r = −0.50, p < 0.001), with moderate-severe NPDR patients having a PLR that was reduced by 22%, on average, compared to the control mean. After correcting for age and dark-adapted baseline pupil size, Tukey adjusted pairwise comparisons indicated significantly smaller melanopsin-mediated PLRs for patients with mild NPDR (t = 5.06, p < 0.0001) and moderate-severe NPDR (t = 6.68, p < 0.0001) compared to the control group.

The patients’ rod-mediated (Fig. 3, middle) PLRs showed less abnormality in the mean response amplitude compared to the differences observed for the melanopsin-mediated condition, but the correlation between disease stage and the rod-mediated PLR was significant (Spearman’s r = −0.28, p = 0.05). However, after correcting for age and the dark-adapted baseline pupil size, Tukey adjusted pairwise comparisons indicated no significant differences in the rod-mediated PLR for any patient group compared to control (all t < 0.69, all p > 0.28).

Figure 3 (bottom) shows that the cone-mediated PLR also decreased with disease stage. There was a significant correlation between disease stage and the cone-mediated PLR (Spearman’s r = −0.37, p = 0.01). After correcting for age and the light-adapted baseline pupil size, Tukey adjusted pairwise comparisons indicated a significant difference in the cone-mediated PLR for patients with moderate-severe NPDR compared to the control group (t = 3.31, p < 0.008). The melanopsin-, rod-, and cone-mediated relative PLRs, adjusted for age and baseline pupil size, are given in Table 3 (rows 1–3).

To evaluate the effects of treatment on the stimulus-driven PLR, sub-analyses were performed after excluding the 8 patients who had focal laser therapy (Table 3; rows 4–6) and after excluding the 19 patients who had received anti-VEGF therapy (Table 3; rows 7–9). Table 3 shows that excluding these patients had negligible effects on the results. Specifically, the results of the sub-analyses were highly similar to the results based on the complete sample, with the exception that excluding patients who had moderate-severe NPDR and a history of anti-VEGF treatment resulted in a non-statistically significant difference from control for the cone-mediated PLR (Table 3; row 9). As noted above, this effect is likely due to the small sample size (N = 5), as approximately 71% of this group had a history of anti-VEGF therapy and were excluded from the sub-analysis.

The relationship between each PLR measure and patient characteristics including age, disease duration, and HbA1c percentage were examined by computing Pearson correlation coefficients. The relationships among these parameters were generally weak (the r values were between −0.33 and 0.15) and were not statistically significant after correcting for multiple correlations. There were also no significant differences between males and females within all subject groups for all PLR measurements (all t < 0.79, all p > 0.43).

A summary of the baseline and PLR abnormalities is provided in Table 4. The two measures (baseline pupil size and PLR) generate four possible outcomes for the patients: (1) abnormal baseline and abnormal PLR; (2) normal baseline and abnormal PLR; (3) abnormal baseline and normal PLR; (4) normal baseline and normal PLR. “Abnormal” refers to values that were below the 5^th^ percentile of the control range and “baseline” refers to the dark-adapted baseline for the melanopsin and rod conditions and the light-adapted baseline for the cone condition. Overall, 71% (12/17) of no the NPDR group, 75% (12/16) of the mild NPDR group, and 94% (16/17) of the moderate-severe NPDR group had an abnormal finding on at least one of the measures. Subtraction of the final column (normal baseline and normal PLR) from 100% provides the percentage of patients within each group who had some measurable abnormality for a given condition. For example, 35% (6/17) of the no NPDR group, 63% (10/16) of the mild NPDR group, and 88% (15/17) of the moderate-severe NPDR group had an abnormal finding under the melanopsin condition alone (i.e. melanopsin-mediated PLR and/or dark-adapted baseline). This finding highlights the potential usefulness of measures performed under rod- and cone-mediated conditions in addition to the more commonly used melanopsin-mediated condition, particularly for diabetic patients who had no NPDR. For these patients, the use of all three measures identified an abnormality in 71% of the sample, whereas the use of the melanopsin condition alone identified an abnormality in 35% of the sample.

## Discussion

The purpose of this study was to determine the effects of diabetic retinopathy on the pupil response measured under conditions designed to target selectively the rod, cone, and melanopsin pathways. Although previous work has shown abnormalities in the light- and dark-adapted steady-state pupil size in individuals who have diabetes^5^,^7^,^36^,^37^, as well as reductions in the melanopsin-mediated PLR^9^, our results extend these findings in two important ways. First, PLRs were recorded under conditions designed to target the rod, cone, and melanopsin pathways allowing for an evaluation of the pupil pathways affected by diabetes. Overall, the results indicate that diabetic patients within all NPDR stages can have abnormalities in the response of the pupil elicited by flashes of light under rod-, cone-, and melanopsin-mediated conditions and that the mean PLR decreases across stage under these conditions. However, statistically significant reductions in the mean PLR, compared to control, were only found for patients who had mild or moderate-severe NPDR under the melanopsin-mediated condition, and for patients with moderate-severe NPDR under the cone-mediated condition. In addition to the analysis of the stimulus-driven PLR, our results extend previous findings of the steady-state (baseline) pupil size by including diabetics who had well-defined disease stages that spanned the NPDR spectrum. The results showed that the mean dark-adapted steady-state pupil size was significantly reduced for all stages, whereas the mean light-adapted steady-state pupil size was reduced significantly only for patients with moderate-severe NPDR.

There is evidence that abnormal sympathetic nervous system innervation might play a role in steady-state pupil abnormalities, but there is also evidence that the steady-state pupil size is significantly constricted in diabetics who have preserved sympathetic innervation of the iris^39^. The results of the present study suggest a relatively small role for an efferent pathway defect due to autonomic deinnervation or stiffness of the iris tissue. If these factors were the primary cause of the abnormalities, then similar abnormalities would be expected under the dark- and light-adapted conditions, which was not the case. For example, diabetics with no and mild NPDR had, on average, a significant deficit only under the dark-adapted condition; an efferent defect would have likely reduced pupil size similarly under the light-adapted condition as well. As an alternative to sympathetic deinnervation, the “equivalent light hypothesis” may provide an explanation for the abnormal dark-adapted steady-state pupil abnormalities. Specifically, this hypothesis proposes that photoreceptor abnormalities lead to continuous activation of the photoreceptor cascade in a manner equivalent to real light^40^. This would be expected to constrict the steady-state pupil in the dark, but have relatively minimal effects on the steady-state pupil in the light, consistent with the pattern of results shown in Fig. 2. Additional work is needed to determine the extent of efferent pathway defects and “equivalent light” on the pupil abnormalities.

Interestingly, the melanopsin-mediated PLR (inferred ipRGC function) was not selectively spared in our diabetic patients, contrary to previous reports of selective ipRGC resistance to injury^41^,^42^ and disease^14^,^43^,^44^. In fact, ipRGC dysfunction appears to be a relatively early indicator of abnormality, as some diabetics who had mild NPDR had melanopsin-mediated PLRs that were outside of the normal range. Melanopsin-mediated PLR abnormalities have also been shown in a previous study of diabetic patients who did not have retinopathy^9^, as well as in patients who have glaucoma^45^,^46^, age-related macular degeneration^47^, and idiopathic intracranial hypertension^24^. Although the PLR defects under the melanopsin-, rod-, and cone-mediated conditions were correlated significantly with the stage of NPDR, defined by the degree of vascular abnormality, there were generally no strong correlations between the PLR and other subject characteristics. This is in contrast to the findings of Feigl *et al*.^9^, who reported a statistically significant relationship between the melanopsin-mediated PLR amplitude and the duration of diabetes. However, diabetes duration can be difficult to estimate and an inability to accurately determine disease duration in our sample may account for the lack of a significant relationship with PLR amplitude. Nevertheless, the weak correlations with standard clinical parameters suggest that the pupil responses may provide insight into the disease beyond that provided by typical clinical measures.

The abnormally constricted steady-state pupil diameters should be considered in the interpretation of the chromatic PLR abnormalities, since a reduced steady-state pupil diameter decreases the amount of light entering the eye. The effects of reduced retinal illuminance can be estimated based on previous reports that quantified the relationship between stimulus luminance and the PLR amplitude^15^,^34^. For example, a reduction in the baseline pupil size from 5.8 mm (the mean of the control group under dark-adapted conditions) to 3.6 mm (the mean of the moderate-severe NPDR group under dark-adapted conditions) would reduce the effective retinal illuminance of the melanopsin stimulus from 4.1 log Td to 3.7 log Td. Based on previous results^15^, this is expected to reduce the relative melanopsin-mediated PLR amplitude by approximately 5.5%. An even smaller reduction (3.3%) is expected under the cone-mediated condition. Thus, attenuation of retinal illuminance due to baseline pupil constriction would have relatively small effects on the reported results. Furthermore, significant reductions in the PLR were found after adjusting for the baseline pupil size in the linear mixed models. The effects of the constricted steady-state pupil size on the ocular optics, other measures of visual function, and on completing tasks of daily living are of interest, but require further study. For example, the reduced retinal illuminance due to the dark-adapted steady-state pupil size reduction could have implications for completing ‘real world’ tasks, such as driving at night.

An additional consideration is that a substantial percentage of patients who had mild or moderate-severe NPDR had a history of treatment with anti-VEGF agents and/or focal laser therapy. However, the effects of these treatments on the PLR and the steady-state pupil size are likely minimal, as shown by the sub-analyses in Tables 2 and 3. This is expected, as the melanopsin- and rod-mediated PLRs are spatially summed responses throughout the entire visual field elicited by a ganzfeld stimulus^32^. As such, local field defects due to focal laser treatment would likely have only a small effect on the PLR. The effects of anti-VEGF agents on pupil function have not been reported, but there is no conclusive evidence of anti-VEGF neurotoxicity^48^,^49^. Our sub-analyses are consistent with a negligible effect of treatment, in that excluding patients who had anti-VEGF or focal laser treatments had minimal effects on the conclusions. However, longitudinal studies that assess the PLR pre- and post-treatment are needed to fully determine potential effects on rod, cone, and melanopsin function.

In summary, we provide evidence that a short-duration, clinically applicable, chromatic pupillometry test can be used to assess neural dysfunction associated with diabetes. Importantly, the results suggest both inner- and outer-retinal dysfunction in some diabetic patients, indicating that pupillometry may be capable of quantifying neural abnormalities in these individuals. Future longitudinal work is needed to evaluate the extent to which pupillometry is useful for identifying individuals at risk of developing severe vascular abnormalities and vision loss.

## Additional Information

**How to cite this article:** Park, J. C. *et al*. Pupillary responses in non-proliferative diabetic retinopathy. *Sci. Rep.* **7**, 44987; doi: 10.1038/srep44987 (2017).

**Publisher's note:** Springer Nature remains neutral with regard to jurisdictional claims in published maps and institutional affiliations.

## Figures and Tables

**Table 1:** Subject characteristics.

	Control (N = 25)	No NPDR (N = 17)	Mild NPDR (N = 16)	Mod-Sev NPDR (N = 17)
Age (yr)	57.0 ± 13.4	58.1 ± 12.0	57.9 ± 8.6	62.0 ± 9.7
Sex	13 M 12 F	5 M 12 F	8 M 8 F	7 M 10 F
Disease duration (yr)		9.0 ± 5.1	16.6 ± 8.3	17.1 ± 10.8
HbA1c (%)		7.0 ± 1.1	8.9 ± 2.3	7.6 ± 1.4
Subjects with focal laser Tx (%)		0	31.3	17.6
Subjects with anti-VEGF Tx (%)		0	43.8	70.6

yr is years; M is male and F is female; HbA1c is glycated hemoglobin; Tx is treatment.

**Table 2:** Mean ( ± standard error of the mean) age-adjusted baseline pupil size (mm).

	Control	No NPDR	Mild NPDR	Mod-Sev NPDR
DA baseline	5.56 ± 0.18	4.48 ± 0.22^*^	4.31 ± 0.22^*^	3.57 ± 0.21^*^
LA baseline	3.62 ± 0.16	3.07 ± 0.19	3.27 ± 0.18	2.77 ± 0.18^†^
**Mean** (**±standard error of the mean**) **age-adjusted baseline pupil size (mm) after excluding patients who had anti-VEGF treatment.**				
DA baseline	5.56 ± 0.18	4.48 ± 0.22^†^	4.23 ± 0.27^*^	3.62 ± 0.24^*^
LA baseline	3.62 ± 0.16	3.07 ± 0.19	3.19 ± 0.23	2.78 ± 0.20^†^
**Mean** (**±standard error of the mean**) **age-adjusted baseline pupil size (mm) after excluding patients who had focal laser treatment**.				
DA baseline	5.56 ± 0.18	4.48 ± 0.22^†^	4.51 ± 0.31^†^	3.71 ± 0.41^*^
LA baseline	3.62 ± 0.16	3.07 ± 0.19	3.44 ± 0.26	2.96 ± 0.36

DA is dark-adapted, LA is light-adapted, ^*^ and ^†^ indicate significant differences from control at the p ≤ 0.001 and p < 0.01 levels, respectively.

**Table 3:** Age and baseline adjusted PLR mean ± standard error of the mean.

	Control	No NPDR	Mild NPDR	Mod-Sev NPDR
Melanopsin	0.41 ± 0.02	0.35 ± 0.02	0.16 ± 0.02^*^	0.14 ± 0.02^*^
Rod	0.19 ± 0.02	0.17 ± 0.02	0.16 ± 0.02	0.13 ± 0.02
Cone	0.28 ± 0.01	0.27 ± 0.01	0.24 ± 0.01	0.22 ± 0.01^†^
**Age and baseline adjusted PLR mean ± standard error of the mean after excluding patients who had focal laser treatment**.				
Melanopsin	0.41 ± 0.02	0.35 ± 0.02	0.28 ± 0.02^*^	0.23 ± 0.02^*^
Rod	0.19 ± 0.02	0.17 ± 0.02	0.17 ± 0.02	0.14 ± 0.02
Cone	0.28 ± 0.01	0.27 ± 0.01	0.24 ± 0.01	0.22 ± 0.01^†^
**Age and baseline adjusted PLR mean ± standard error of the mean after excluding patients who had anti-VEGF treatment**.				
Melanopsin	0.41 ± 0.02	0.35 ± 0.02	0.28 ± 0.03^*^	0.17 ± 0.03^*^
Rod	0.19 ± 0.02	0.17 ± 0.02	0.17 ± 0.03	0.11 ± 0.03
Cone	0.28 ± 0.01	0.27 ± 0.01	0.27 ± 0.02	0.22 ± 0.02

^*^ and ^†^ indicate significant differences from control at the p ≤ 0.001 and p < 0.01 levels, respectively.

**Table 4:** Summary of baseline and PLR abnormalities.

		Abnormal baseline Abnormal PLR	Normal baseline Abnormal PLR	Abnormal baseline Normal PLR	Normal baseline Normal PLR
Melanopsin	No NPDR	6%	0%	29%	65%
	Mild NPDR	38%	6%	19%	38%
	Mod-Sev NPDR	53%	6%	29%	12%
Rod	No NPDR	6%	12%	35%	47%
	Mild NPDR	19%	6%	44%	31%
	Mod-Sev NPDR	35%	12%	47%	6%
Cone	No NPDR	12%	0%	35%	53%
	Mild NPDR	13%	19%	6%	63%
	Mod-Sev NPDR	29%	12%	29%	29%
